# *In vitro* Studies on The Inhibition of Replication of Zika and Chikungunya Viruses by Dolastane Isolated from Seaweed *Canistrocarpus cervicornis*

**DOI:** 10.1038/s41598-020-65357-7

**Published:** 2020-05-19

**Authors:** Claudio Cesar Cirne-Santos, Caroline de Souza Barros, Mariana Cavalcante de Oliveira, Vitor Won-Held Rabelo, Renata Campos Azevedo, Valeria Laneuville Teixeira, Davis Fernandes Ferreira, Izabel Christina Nunes de Palmer Paixão

**Affiliations:** 1 Laboratório de Virologia Molecular e Biotecnologia Marinha, Programa de Pós-graduação em Ciências e Biotecnologia, Departamento de Biologia Celular e Molecular, Instituto de Biologia, Universidade Federal Fluminense, Niterói, Brazil; 2 Laboratório Produtos Naturais de Algas Marinhas (ALGAMAR), Departamento de Biologia Marinha, Instituto de Biologia, Universidade Federal Fluminense, Niterói, Brazil; 3 Instituto de Microbiologia, Departamento de Virologia, Universidade Federal do Rio de Janeiro, Rio de Janeiro, Brazil; 4 Laboratório de Biologia e Taxonomia de Algas (LABIOTAL), Programa de Pós-graduação em Biodiversidade Neotropical, Instituto de Biociencias, universidade Federal do Estado do Rio de Janeiro, Rio de Janeiro, RJ Brasil

**Keywords:** Biologics, Drug screening, Viral infection

## Abstract

The lack of vaccines and antiviral treatment, along with the increasing number of cases of Zika virus (ZIKV) and Chikungunya virus (CHIKV) infections, emphasize the need for searching for new therapeutic strategies. In this context, the marine brown seaweed *Canistrocarpus cervicornis* has been proved to hold great antiviral potential. Hence, the aim of this work was to evaluate the anti-ZIKV and anti-CHIKV activity of a marine dolastane isolated from brown seaweed *C. cervicornis* and its crude extract. Vero cells were used in antiviral assays, submitted to ZIKV and CHIKV, and treated with different concentrations of *C. cervicornis* extract or dolastane. The crude extract of *C. cervicornis* showed inhibitory activities for both ZIKV and CHIKV, with EC_50_ values of 3.3 μg/mL and 3.1 μg/mL, respectively. However, the isolated dolastane showed a more significant and promising inhibitory effect (EC_50_ = 0.95 µM for ZIKV and 1.3 µM for CHIKV) when compared to both the crude extract and ribavirin, which was used as control. Also, the dolastane showed a very potent virucidal activity against CHIKV and was able to inhibit around 90% of the virus infectivity at 10 μM. For the ZIKV, the effects were somewhat lower, although interesting, at approximately 64% in this same concentration. Further, we observed that both the extract and the dolastane were able to inhibit the replication of ZIKV and CHIKV at different times of addition post-infection, remaining efficient even if added after 8 hours post-infection, but declining soon after. A synergistic effect using sub-doses of the extract and isolates was associated with ribavirin, inhibiting above 80% replication even at the lowest concentrations. Therefore, this work has unveiled the anti-ZIKV and CHIKV potential of *C. cervicornis* crude extract and an isolated dolastane, which, in turn, can be used as a preventive or therapeutic strategy in the future.

## Introduction

Arthropod-borne viruses, mainly described as arboviruses, pose a significant threat to human and animal health in many parts of the world. Clearly, it is now known that such pathogens are transmitted by arthropod vectors, such as mosquitoes and ticks, to susceptible vertebrates. In addition, they are targets of studies and precautions due to the possibility of emerging and reemerging virus infections^1^. Humans are mostly incidental hosts because they do not produce significant viremia and do not contribute to the transmission cycle. Thus, it is observed that they acquire the viral infection during the blood-feeding process of an infected vector and that mosquitoes are the main vector for most arboviruses^2,3^.

The infection mechanisms of arboviruses are vastly classified. They can lead to central nervous system impairment with compromises ranging from mild to paralytic and death processes, as well as hemorrhagic lesions, fever, capillary leakage, shock, jaundice, liver damage, and culminating in death. In addition, polyarthritis and rash may be common. However, a large number of these infections presents asymptomatically or may result in nonspecific symptoms^4,5^.

The Zika virus (ZIKV), so named in reference to the Ugandan forest, was first isolated in 1947 and is a flavivirus closely related to dengue. It was initially recognized as causing an asymptomatic infection or producing a febrile disease in humans and, for decades, did not express major public health concerns^6^. However, several cases of microcephaly were initially linked to the virus. Physicians working in the Northeast region of Brazil have detected a significant increase in the incidence of children being born with microcephaly following the identification of virus entry in the country. The discovery of the possible transmission of the virus through sexual contact or secretions (saliva, urine), and the absence of vaccines or specific treatments have been determinant for a high level of worldwide concern. However, diagnostic confirmation has been difficult due to the lack of markers or tests that have real diagnostic capacity, leading to constant false-positive cases^7,8^. In addition, during the outbreak in French Polynesia, it was observed that 42 patients with symptoms of ZIKV infection presented Guillain-Barré syndrome, which represented a considerable increase in the number of cases. These observations had already been related to flavivirus at another time, but not to ZIKV infection. Studies have associated ZIKV infection with fetal microcephaly, and it was declared by the WHO in 2016 as the “public health emergency of international concern”^9^. However, the relationship between intrauterine infection by ZIKV and recent cases of microcephaly has been panicky for many women at any gestational age.

The Chikungunya virus (CHIKV) is a virus transmitted by arthropods to humans mainly by the mosquito Aedes aegypti. The virus was primarily isolated in Tanzania in 1952 and for years has been reported in sporadic cases and several outbreaks. To date, reports of CHIKV infection were obtained in several African countries, in the Indian subcontinent, and Southeast Asia. In recent years, many outbreak cases have been reported in a large geographical area that includes African islands in the Indian Ocean and Indian subcontinent. Studies have shown that in August 2015, the autochthonous transmission was detected in 33 countries and territories of the Americas, and Latin America registered almost one million cases^10^. Severe forms of CHIKV infection appear to be associated with multiple organ failure, hepatitis, meningitis, nephritis, encephalitis, bullous dermatitis, myocarditis, and cardiac arrhythmias. However, studies show that although the most severe or atypical manifestations of CHIKV infection are uncommon, except for severe arthralgia, the overall mortality rate of these complications has remained quite high.

Previous studies have shown the antiviral potential of *C. cervicornis* against HSV-1 *in vitro* assays^11^ and also its therapeutic efficacy in BALB/c mice infected with the virus and treated with an extract ointment^12^. We also demonstrated the potential of two dolastanes and a secodolastane isolated from *C. cervicornis* against HIV-1^13^.

## Experimental Section

### Seaweed material

Specimens of brown seaweed *C. cervicornis* (Kützing) De Paula and De Clerck were collected at Praia do Velho in Angra dos Reis, in the south of Rio de Janeiro State, Brazil (lat. 23°01′S, long. 44° 00′W). Voucher specimens (HRJ 10754) were deposited in the Herbarium of the Universidade do Estado do Rio de Janeiro (UERJ). The seaweeds were washed with local seawater and separated from sediments, epiphytes, and other associated organisms.

### Extraction

**Figure 1:**
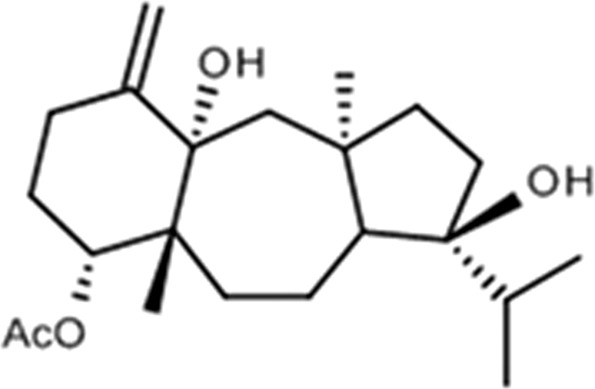
Chemical structure of compound 1 from *Canistrocarpus cervicornis*.

In order to prepare the crude extract, air-dried seaweeds were exhaustively extracted with CH_2_Cl_2_ at room temperature. In previous studies, the use of CH_2_Cl_2_ extracts most of the diterpenes from the algae. The extract was evaporated under reduced pressure, yielding a brownish residue. The crude extract was acetylated to increase the amount and stability of the most abundant diterpenes by replacing the secondary hydroxyl groups by acetyl groups as described previously by our group^14^. The acetylated extract was subjected to silica gel chromatography (3 × 40 cm) eluted with 100% n-hexane to 100% EtOAc, in steps of 10% and 100 mL, from which 44 fractions were obtained. The fractionation was monitored by thin-layer chromatography, and spots of diterpenes were detected as pink-colored spots after heating the plated specimens at 100 °C for 3 minutes. The fractions 26, 27, and 28 yielded the dolastane diterpene (4R,9S,14S)-4α-acetoxy-9β,14α-dihidroxydolast-1(15),7-diene^14^. The structure of the diterpene was assigned by comparison of physical and spectroscopic data with reported values^15^ (Fig. 1).

### Cells and virus

Vero cells (African green monkey kidney) were grown in Dulbecco’s Modified Eagle Medium (DMEM; Invitrogen, cat. no 11960) supplemented with 5% Fetal Bovine Serum (FBS; Invitrogen), 2 mmol L^−1^ L-glutamine (Invitrogen, cat. no. 25030). Antibiotics were added at a final concentration of 50 units/mL penicillin and streptomycin (Invitrogen, cat. no. 15070). ZIKV (ATCC® VR-1839™) was amplified in C6/36 mosquito cells line from *Aedes albopictus*, adapted to grow at 28 °C, was cultured in L-15 Medium (Leibovitz) supplemented with 0.5% tryptose phosphate broth, 0.03% glutamine, 1% MEM non-essential amino acids solution and 5% FBS. CHIKV experiments were performed with an isolated virus of infected patients, and the sequence was deposited in GenBank under accession number: MK910738 (BRA/RJ/1 F). The manipulation was carried out in a Safety Laboratory, level 3 risk classification, as determined by the Ministry of Health, Ordinance No. 2,349, of September 142017.

### Effect of the compounds on the cellular viability

Cytotoxicity of the seaweed crude extract and dolastane in Vero cells was tested *in vitro* using the MTT method^16^. Briefly, 2 × 10^4^ Vero cells were seeded into each well of a 96 well plate and incubated for 24 hours (h) at 37^o^C with different increasing concentrations ranging from 50 to 1000 μg/mL and 50 to 1000 µM of extract and dolastane, respectively, diluted in DMEM. After this period, the cells were submitted to the MTT method and evaluated on a microplate reader for the determination of cell viability as described in the method. Cells treated with DMSO (1.0%) in DMEM were used as control. Relative cell viability was calculated as a percentage comparing treated cells with the untreated cells and the cells in DMSO. Concentrations that maintained more than 90% of cell viability after treatment were considered non-toxic.

### TCID50 assay for infectious virus

To assess whether the compounds affect replication of ZIKV and CHIKV, 2 × 10^5^ Vero cells were seeded into each well of a 24-well plate the day before infection. The virus inoculum was diluted to a multiplicity of infection (MOI) of 0.1 in serum-free DMEM. After 1 h of adsorption at 37 °C, the viral inoculum was removed by aspiration, the cells were washed with PBS to remove the non-adsorbed viral residual, and fresh medium was added at the indicated concentration of the compounds. Infected cells were also treated with culture medium containing 1% DMSO to determine the residual antiviral effect, and ribavirin was used as a positive control for antiviral activity. After 24 h of incubation at 37 ^o^C, the culture medium was collected, and the yield of the infectious virus in the cell supernatant was determined by titration using the TCID50 assay in VERO cells and was also evaluated by assay for the production of viral plaques.

### Time-of-Drug-addition assay

To initially understand the mechanism of action of the *C. cervicornis* extract and dolastante, a time-of-drug-addition assay was carried out. Monolayers of Vero cells grown in 24-well plates were infected with ZIKV and CHIKV at an MOI of 3 at time zero. Cells were treated with the extract or dolastane at a final concentration of 10 μg/mL and 10 µM, respectively, at the following times: 0, 1, 2, 4, 6, and 8 h after virus infection. After a 12 h incubation, the supernatants were collected and added to naive cells. Viruses adsorption was allowed for 2 h; then cells were washed and covered with 2.5% carboxymethylcellulose in culture medium. After 72 h of incubation at 37 ^o^C, the cells were fixed with 20% formaldehyde for 2 h and stained with crystal violet for 5 minutes. Plates formed after each treatment were counted and titer calculated.

### Determination of virucidal activity

To assess whether dolastane and *C. cervicornis* extract have a direct effect on the infectivity of ZIKV and CHIKV, approximately 200 plaque-forming units of the viruses were incubated at 37^o^C in DMEM containing both compounds at three indicated concentrations (5, 10 and 20 μg/mL for extract and 5, 10 and 20 µM for dolastane). In order to the virus adsorption to occur, cells were incubated for 1 h and then washed for removal of the residual virus. After the incubation time, the mixture of virus and compounds was diluted 10 times; that way, the concentration was outside the inhibition range. DMSO 1% and ribavirin were used as controls. Virus titer was determined by plaque assays, as abovementioned.

### Evaluation of the combinatorial effect of the compounds

To assess whether the combined compounds show increased antiviral effects, more directly described as a synergistic effect, VERO cells were infected and treated with the ZIKV and CHIKV viruses with MOI of 0.1 and incubated for 2 h, then the compounds were added separately at different concentrations and 0.5 µM for dolastane and Ribavirin and 0.5 μg/mLfor the extract (doses with a low inhibitory effect <20%). In addition, they were also added at higher doses like 10 µM for dolastane and ribavirin and 10 μg/mL for the extract (doses with high inhibitory effect > 80%). In other wells, the compounds were added combined (dolastane plus Ribavirin and Extract plus Ribavirin) in low and high doses, as indicated above. After the plates were washed and carboxymethyl cellulose (CMC) was added for evaluation of viral plaque formation. Cells were fixed and stained after 48–72 h, and viral plaques were counted. Additionally, the results were analyzed by the Loewe and Bliss synergy and antagonism methods using Combenefit software, for the determination of synergism^17^.

### Statistical analysis

The data were analyzed by one-way analysis of variance (ANOVA) followed by Tukey post-test using the GraphPad Instat version 3 program. A p-value of <0.05 was considered statistically significant. In the case of the Synergism results, the T-test was used for the best determination of significance.

## Results

### Effect of Dolastane and extract of *C. cervicornis* on cell viability

Cell viability in the presence of *C. cervicornis* extract and dolastane was evaluated using the MTT method. As a comparison, we used broad-spectrum antiviral ribavirin, which has also been shown to have antiviral activity against both ZIKV and CHIKV. The compounds added at increasing concentrations showed a low dose toxicity profile, and besides, the compounds showed less toxicity than ribavirin. The compounds maintained cell viability above 80% at concentrations up to 200 μg/mL for the extract and 200 μM for dolastane, declining at higher concentrations (data not shown). The CC_50_ values presented by the compounds were high for both *C. cervicornis* extract (438 μg/mL) and dolastane (935 μM), and ribavirin showed higher cytotoxicity than the compounds (297 µg.mL^−1^). Therefore, lower concentrations than those reported above for each drug were selected for the virus production assay (Table 1).

**Table 1:** Cytotoxicity (CC_50_), anti-ZIKV, or anti-CHIKV profile (EC_50_) and selectivity index (SI) of dolastane diterpenes.

Compounds		ZIKV		CHIKV	
	**CC**_**50**_^**a**^(μg or μM)	EC_50_^b^(μg or μM)	SI^c^	EC_50_^b^(μg or μM) SI^c^	
Extract	438 μg/mL ± 5.18	2.15 ± 0.22	203	2.45 ± 1.02	178
Dolastane	935 µM/mL ± 11.0	0.75 ± 0.18	1246	1.28 ± 0.47	730
Ribavirin	297 μM/mL ± 4.25	3.95 ± 0.95	75.2	2.42 ± 0.49	122

The mean values ± standard deviations are representative of three independent experiments.

^a^Concentration required to reduce 50% cell viability when compared to untreated controls.

^b^Concentration that reduced 50% of ZIKV or CHIKV replication when compared to infected and untreated controls.

^c^Selectivity index was defined as the ratio between CC_50_ and EC_50_.

### Virus yield assay

**Figure 2:**
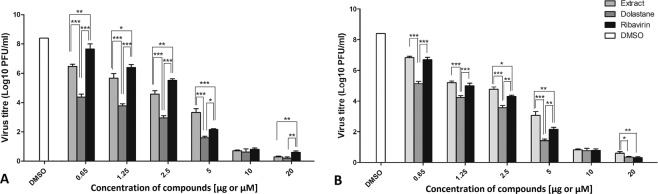
Inhibition of ZIKV or CHIKV replication by *C. cervicornis* extract, dolastane, and ribavirin. (**A**) Inhibition of ZIKV replication. (**B**) Inhibition of CHIKV replication. Vero cells were infected with ZIKV or CHIKV (MOI 0.1) and treated at concentrations of 0.65, 1.25, 2.5, 5, 10, or 20 μg/mL or µM. The results were evaluated from three independent experiments in triplicate. Data are presented as the percentage of virus titer when compared to control cells and are expressed as the mean of three experiments ± standard error. Statistical analysis was performed using the Tukey test in comparison to *C. cervicornis* extract, dolastane, and ribavirin in each concentration: *p < 0.05; **p < 0.01; ***p < 0.001.

The evaluation of the antiviral effect was performed on Vero cells. Vero cells were infected with ZIKV and CHIKV using a multiplicity of infection of 0.1 and treated for 24 h with increasing concentrations of the substances. The TCID50 method was used to titrate the virus yield, and the concentration capable of reducing virus yield by 50% (EC_50_) was obtained from a dose-response plot and is shown in Fig. 2A,B. Ribavirin was used as control. The results showed that concentrations of the compounds ranging from 0.65 to 20 μM led to a significant reduction in infectious viral yields when compared to the treatment. The data demonstrate a dose-response curve, reaching about 90% inhibition or more than 5-fold reduced virus production for both viruses. The relationship between the cytotoxicity and the antiviral effect of the compounds was evaluated by means of the Selectivity Index - SI (ratio CC_50_/EC_50_) and determined for each molecule, obtaining unitless values. Our results show that dolastane presented an expressive SI for both ZIKV equal to 1246 and CHIKV equal to 730, proving to be much higher than the extract of *C. cervicornis* and Ribavirin as can be observed in Table 1.

### Time-of-drug-addition assay

**Figure 3:**
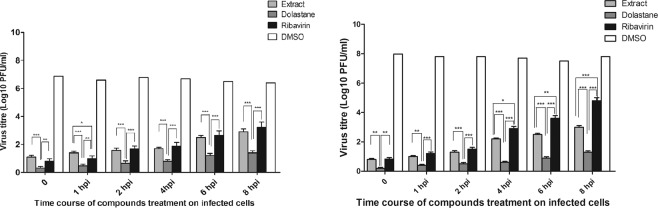
Effect of the addition of *C. cervicornis* extract, dolastane, and ribavirin on ZIKV or CHIKV replication over time. (**A**) Inhibition of ZIKV replication. (**B**) Inhibition of CHIKV replication. Monolayers of Vero cells were infected with ZIKV or CHIKV at an MOI of 3 at time zero. At times indicated, extract, dolastane or ribavirin was added to a final concentration of 10 μg/mL for extract or 10 μM/mL, for dolastane or ribavirin. The data were presented by the virus titers in PFU/mL when compared to control cells and are expressed as the mean of three experiments ± standard error. Statistical analysis was performed using the Tukey test in comparison to *C. cervicornis* extract, dolastane, and ribavirin in each concentration: *p < 0.05; **p < 0.01; ***p < 0.001.

In the time-of-addition assay, we observed that the extract and the dolastane had a very potent inhibitory effect with a 4 or 6-fold decreased virus production for both viruses when added at time zero (simultaneous treatment) (Fig. 3A,B). The effects were observed in post-treatment by inhibition of plaque assay for CHIKV and ZIKV in addition to the determination of viral inhibition by TCID50. As can be seen, the inhibitory potential for the compounds was quite significant with virus inhibition of more than 3 Log_10_. The dolastane effects on ZIKV presented better results when compared to ribavirin, demonstrating to be a potent inhibitor up to the eighth h of post-treatment (Fig. 3A). On the CHIKV inhibiting front, dolastane presents a potent inhibition of viral replication for up to 8 h, while ribavirin significantly reduces its inhibitory potential within 4 h of post-treatment, and the extract behaves similarly to ribavirin (Fig. 3B).

### Determination of the virucidal activity of the compounds

**Figure 4:**
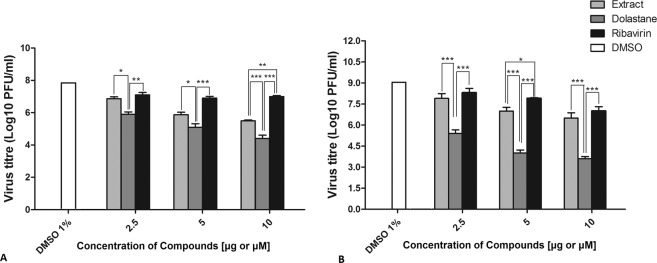
Virucidal effect. (**A**) Effect on the ZIKV. (**B**) Effect on the CHIKV. The viral suspension (ZIKV or CHIKV) was incubated with *C. cervicornis* extract, dolastane, and ribavirin at concentrations of 2.5, 5, and 10 µg/mL for extract or 10 µM for dolastane or ribavirin for 2 h and then added to Vero cells. Viral cytopathic effect was assessed after 72 h of incubation. Data are presented as the percentage of virus titer when compared to control cells and are expressed as the mean of three experiments ± standard error. Statistical analysis was performed using the Tukey test in comparison to *C. cervicornis* extract, dolastane, and ribavirin in each concentration: *p < 0.05; **p < 0.01; ***p < 0.001.

The virucidal activity assay was performed using Vero cells to explore whether the inactivation of the virus particle is a mechanism of action of the studied products. Virus titer was determined by plaque assay and TCID50. The *C. cervicornis* extract inhibited the replication of ZIKV and CHIKV in a dose-dependent manner. However, we observed an inhibition of ZIKV replication up to 40% or (1 Log_10_ inhibition) in contrast to the inhibition of CHIKV that was able to inhibit up to 60% or (1 Log_10_ inhibition) at the concentration of 10 µM used (Fig. 4a,b). On the other hand, the dolastane exhibited more potent effects against both viruses, inhibiting nearly 2 to 3 Logs to the production of ZIKV and CHIKV, respectively, at 10 µM. Ribavirin showed weak virucidal activity, decreasing up to 1 log_10_ of ZIKV and CHIKV titers.

### Evaluation of the synergistic effect of compounds with Ribavirin

**Figure 5:**
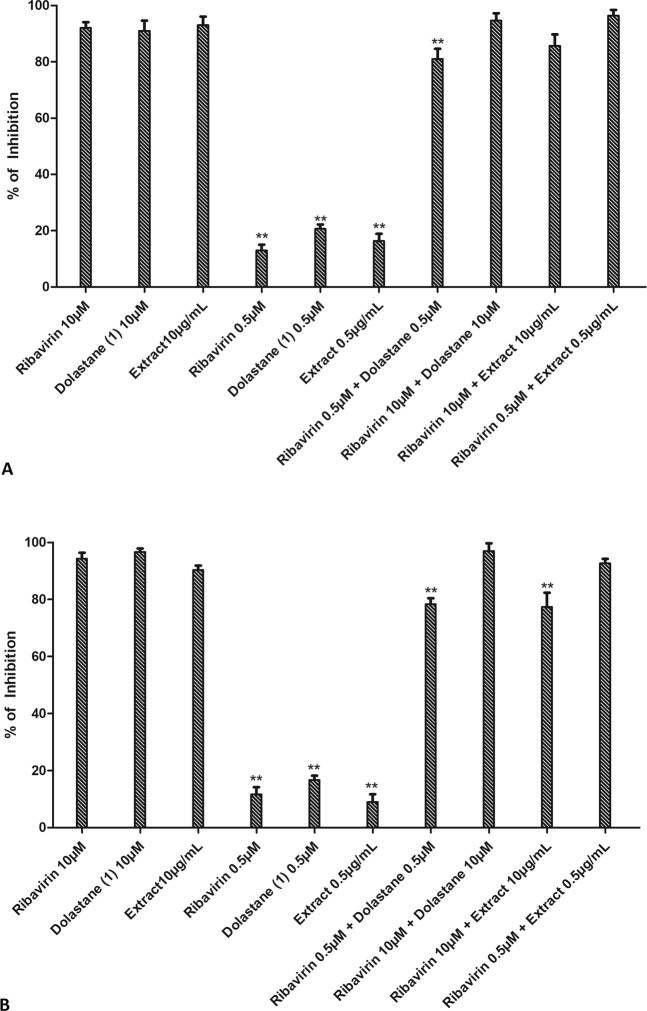
Evaluation of the synergistic effect in combination of *C. cervicornis* extract or dolastane with ribavirin on ZIKV or CHIKV replication. (**A**) Inhibition of ZIKV replication. (**B**) Inhibition of CHIKV replication. Monolayers of Vero cells were infected with ZIKV at an MOI of 0.1 and subsequently treated with extract and Ribavirin sub-doses (0.5 µg/mL and 0.5 µM, respectively) and concentrations of 10 µg/mL and 10 µM. In addition, the dose combination was also performed at both concentrations for synergism assessment. Evaluation of the synergistic antiviral effect was determined by inhibition of cytopathic effect by plaque assay. Data are presented as the percentage of virus titer when compared to control cells and are expressed as the mean of three experiments ± standard error. Statistical significance following a one-sample t-test is indicated (*p < 0.05; **p < 0.001).

**Figure 6:**
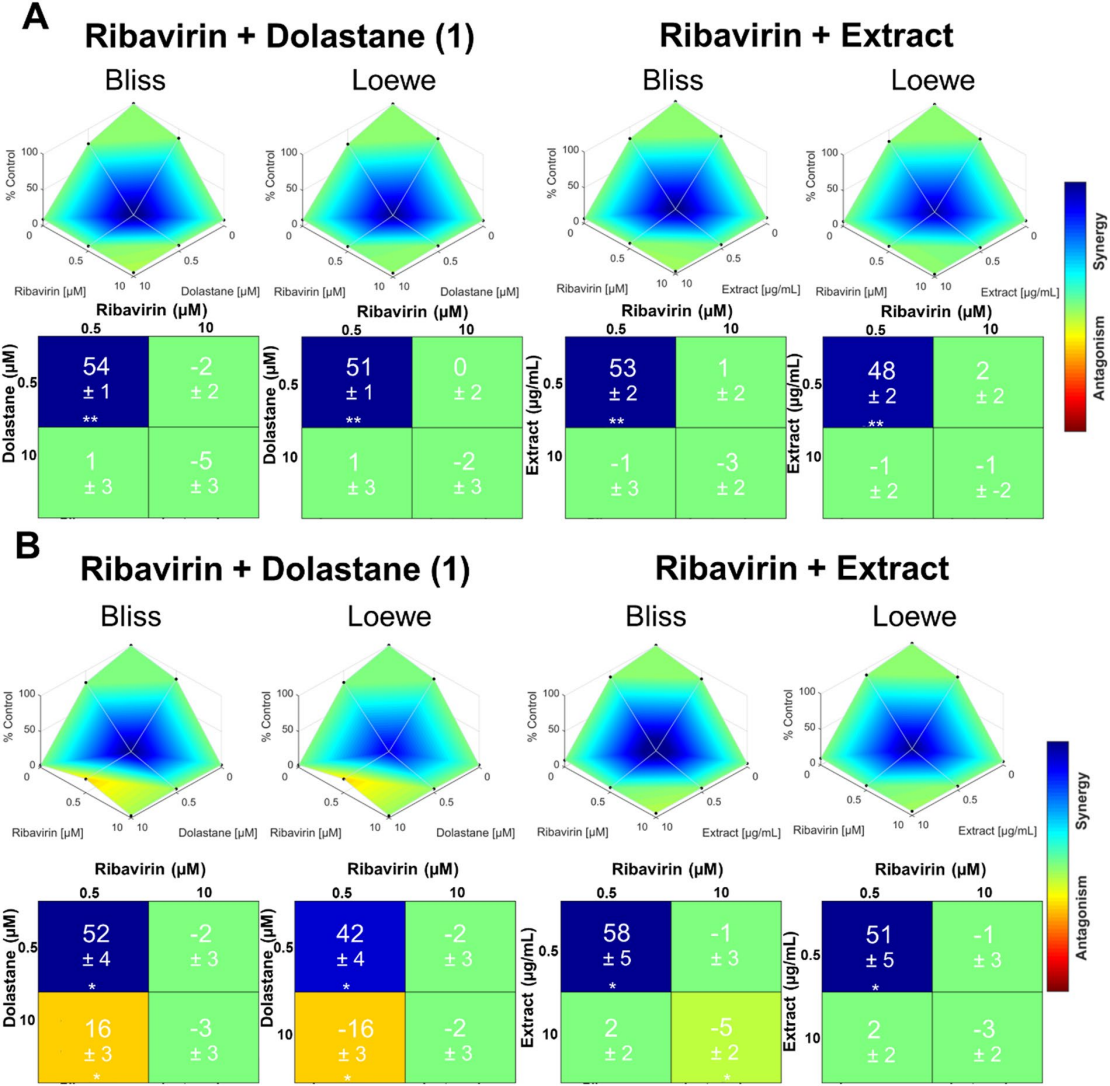
Synergistic effects of ribavirin and extract or dolastane against ZIKV and CHIKV. Synergy distribution plots and score matrix tables calculated by Bliss and Loewe methods for combination effects of ribavirin and extract or dolastane against (**A**) ZIKV, and (**B**) CHIKV are shown. Synergy scores are represented as mean ± standard deviation (n = 3). Statistical significance following a one-sample t-test is indicated (*p < 0.05; **p < 0.001).

The determination of the synergistic effect may be an essential strategy for the use of drugs. For this, the antiviral effects were initially determined at concentrations previously studied with low inhibitory potential (0.5 μg/mL extract or 0.5 μM dolastane and ribavirin), which showed replication inhibition rates lower than 20% at these concentrations. Also, they were used separately at higher inhibition concentrations (10 μg/mL extract or 10 μM dolastane and ribavirin), which were able to inhibit above 90% of viral replication. The compounds were then combined at the lower concentrations (extract 0.5 μg/mL plus 0.5 μM ribavirin) and at higher concentrations (10 µg.mL^−1^ extract plus 10 μM Ribavirin). In Fig. 5A,B, we can observe that the association of the lower concentrations of both *C. cervicornis* extract with Ribavirin and Dolastane with Ribavirin was able to inhibit up to 80% to 90% of viral replication for both ZIKV and or CHIKV. These effects were similar to the ones observed of the combination of the compounds with Ribavirin at the highest concentrations that were between 80 and 90%. In light of the limitations of individual synergy models^18^, we employed a consensus strategy by combining results from both Loewe and Bliss synergy and antagonism methods. Interestingly, the combination of ribavirin (0.5 µM) with the extract (0.5 μg/mL) or the isolated dolastane (0.5 µM) resulted in a substantial synergy effect for both ZIKV and CHIKV, with an increased effect of 52–58% and 42–51% according to Bliss and Loewe models, respectively. In contrast, other combinations showed mostly additive effects (Figure Fig. 6A,B). Most surprisingly, we noticed an antagonism effect for the combination of ribavirin (0.5 µM) and dolastane (10 µM) against CHIKV.

## Discussion

In the last years, the rapid widespread of ZIKV and CHIKV infections worldwide^7,19^ associated with severe manifestations and high impact on public health care and quality of patients’ life have led to the search for new antiviral compounds to tackle these diseases^20,21^. Marine organisms, such as seaweeds, have great potential as sources of drug candidates with low toxicity and high antiviral activity against several viruses^22,23^. Herein, we isolated a dolastane from the seaweed *C. cervicornis* and evaluated the compounds and the crude seaweed extract against ZIKV and CHIKV.

Both products had an interesting bioactivity profile. For instance, they exhibited potent antiviral activity with EC_50_ values of 2.15 μg/mL and 0.75 µM for the extract and the isolated compound, respectively, against ZIKV replication. Likewise, both products were able to inhibit CHIKV replication with EC_50_ values of 2.45 µg.mL^−1^ for the extract and 1.28 μM for the dolastane. Interestingly, low cytotoxicity was observed for the dolastane (CC_50_ = 935 μM) and the crude extract (CC_50_ = 438 μg/mL), resulting in promising SI for the extract (203 and 178 for ZIKV and CHIKV, respectively) and the dolastane (1246 and 730 for ZIKV and CHIKV, respectively). These data are representative when we consider that the dolastane, which showed the best antiviral potential, is a compound isolated from the *C. cervicornis* seaweed that displays an interesting anti-ZIKV and anti-CHIKV activity, similar to that observed for the other compounds tested, but with high inhibitory potential^24–26^.

Investigating the mechanism of action of compounds is an essential step in drug development and may help in the evaluation of their promising and specificity profiles. In this scenario, finding compounds with low toxicity and that inhibit different steps of viral replication may be important to determine whether their clinical use is feasible in the future^27^. Thereby, we evaluated the inhibitory effect of the products added at different times after viral infection. Interestingly, the dolastane maintained its inhibitory potential above 80% against CHIKV even when added at 24 h post-infection while the similar effects were observed against ZIKV when treated after up to 16 h post-infection. Our studies demonstrate that it may be feasible to search for new compounds capable of acting on viral replication at different stages and increasing the prospect of discovering new drugs for clinical use similar to that occurring for the treatment of HIV infection^28^, herpes^29^, hepatitis^30^ or even to more than one viral treatment^31,32^. Previously, we performed time-of-drug-addition studies against ZIKV with the viral polymerase inhibitor 7DMA, considering only post-treatment at different time points up to 24 h post-infection. They showed that the addition of the compound to infected cells could be delayed up to ~10 h post-infection without much loss of antiviral efficacy, which suggests these compounds may act at both pre-entry and post-entry stages of infection.

Another approach widely explored for tackling viral diseases is the development of preventive strategies such as products that inactivate the viral particle before entering into host cells^33^. We observed that dolastane showed a dose-dependent virucidal activity, inhibiting up to 4 Log_10_ the CHIKV production (Fig. 4A,B). However, although the compounds exhibit some virucidal activity, this moderate expression can inhibit up to 3 Log_10_ of viral particle infectivity in the highest concentration tested. In the preparation of preventive measures on the activity of some viral infections, many different working groups are aiming to act directly on the viral particle preventing the appearance of symptoms or even reducing the morbidity and mortality caused by the infection^34^. Thus, these compounds have therapeutic potential, as it acts directly on the virus particle. This effect is significant due to the possible sexual transmission of ZIKV^35^. In this sense, this extract has potential as a preventive microbicide for preventing the transmission of the virus through sexual intercourse^36^.

Combining different drugs has proved to be an interesting strategy to increase the desired biological activity considerably with a decreased toxicity, as already proposed by some research groups^37,38^. In our studies, we evaluated the combination of the tested compounds, both the *C. cervicornis* extract and the isolated dolastane combined with ribavirin, both at low concentrations, *i.e*., concentrations that were not able to expressively inhibit the replication of ZIKV or CHIKV, inhibiting only up to 20% of viral replication if added separately. Our results demonstrated that the combination determined a strong synergism in both inhibition of ZIKV (Fig. 5) and inhibition of CHIKV (Fig. 6), reaching over 90% inhibition of replication when combined, which may suggest that the combined use can be an important treatment strategy, bringing lower risk to people’s health.

## Conclusions

Our findings showed that the crude extract of seaweed *C. cervicornis* tested showed activity against ZIKV and CHIKV. Also, the dolastane extracted from these algae has shown an even more expressive result, demonstrating that marine algae are an interesting source for drug discovery and the development of novel anti-ZIKV and anti-CHIKV agents. Furthermore, upcoming studies focused on the specific mechanism of action aspects and *in vivo* experiments may corroborate the importance of this compound and its potential for reducing morbidity and mortality of infections caused by these viruses.
